# Vitamin C Transporters and Their Implications in Carcinogenesis

**DOI:** 10.3390/nu12123869

**Published:** 2020-12-18

**Authors:** Kinga Linowiecka, Marek Foksinski, Anna A. Brożyna

**Affiliations:** 1 Department of Human Biology, Faculty of Biological and Veterinary Sciences, Nicolaus Copernicus University, 87-100 Toruń, Poland; 2 Department of Clinical Biochemistry, Faculty of Pharmacy, Collegium Medicum, Nicolaus Copernicus University, 85-092 Bydgoszcz, Poland

**Keywords:** vitamin C, SVCT polymorphisms, carcinogenesis

## Abstract

Vitamin C is implicated in various bodily functions due to its unique properties in redox homeostasis. Moreover, vitamin C also plays a great role in restoring the activity of 2-oxoglutarate and Fe^2+^ dependent dioxygenases (2-OGDD), which are involved in active DNA demethylation (TET proteins), the demethylation of histones, and hypoxia processes. Therefore, vitamin C may be engaged in the regulation of gene expression or in a hypoxic state. Hence, vitamin C has acquired great interest for its plausible effects on cancer treatment. Since its conceptualization, the role of vitamin C in cancer therapy has been a controversial and disputed issue. Vitamin C is transferred to the cells with sodium dependent transporters (SVCTs) and glucose transporters (GLUT). However, it is unknown whether the impaired function of these transporters may lead to carcinogenesis and tumor progression. Notably, previous studies have identified SVCTs’ polymorphisms or their altered expression in some types of cancer. This review discusses the potential effects of vitamin C and the impaired SVCT function in cancers. The variations in vitamin C transporter genes may regulate the active transport of vitamin C, and therefore have an impact on cancer risk, but further studies are needed to thoroughly elucidate their involvement in cancer biology.

## 1. Vitamin C Properties and Redox Homeostasis

**Figure 1:**
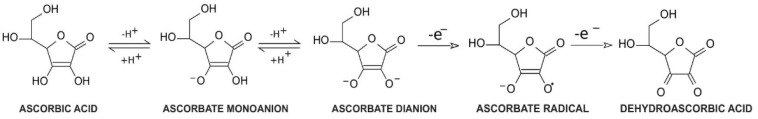
Vitamin C redox states. Ascorbic acid undergoes two reversible hydrogen dissociations to form ascorbate monoanions and ascorbate dianions, respectively. Then, ascorbate dianons can be subsequently subjected to one-electron oxidation to create ascorbate radicals. These are not highly reactive components; however, they can undergo another one-electron oxidation to form dehydroascorbic acid.

Vitamin C, a common name for l-ascorbic acid or l-ascorbate, is a six-carbon lactone. It can be synthesized from glucose by enzyme l-gulono-1,4-lactone oxidase, which can be found in almost every mammal’s liver, with the exception of humans, other primates, bats, and guinea pigs [1,2]. Consequently, these species must provide their organisms with l-ascorbate exogenously. Since l-ascorbate is a key factor in intensifying the activity of numerous enzymes located throughout the human body, the external intake of vitamin C is particularly important. Its unique properties are connected with its structure and biochemistry: ascorbic acid can be transformed into ascorbate monoanion or ascorbate dianion via the dissociation of one or two hydrogen ions from hydroxyl groups located at carbon 2 and carbon 3. In physiological pH ascorbate functions as a monoanion, which is its primary form. Ascorbic acid is known to be a significant reducing factor, easily subject to the compilations of two one-electron oxidations. The first one is accomplished via ascorbate radical generation [3], providing a specific chemical structure (resonance stabilization) with a stable yet not highly reactive form [4]. The ascorbate radical can then undergo the second one-electron oxidation to form dehydroascorbic acid (DHA) (Figure 1) [3]. This ability makes ascorbate a particularly good defender against other free radicals by substituting them with more stable and less reactive compounds [4]. Furthermore, in vitro studies demonstrated that an increased level of ascorbate can act as a stimulus to cooperate with superoxide dismutase in the removal of superoxides [5]. DHA and ascorbate radicals can also be reduced to ascorbate in a reversible manner [3]. 

Despite its ability to act as a reducing agent, ascorbate can also perform a key role as an oxidative factor. Although the oxidation process of ascorbate occurs mainly in the presence of catalytic metals, the process can be performed by ascorbate itself (autooxidation) but at a significantly slower rate in neutral pH [6]. The effect of vitamin C oxidation is hydrogen peroxide formation, which can affect cellular metabolism, by changing intracellular redox stability [6,7]. Moreover, vitamin C can act as a prodrug through its ability to serve as a reducing and oxidizing factor [7]. The privileged form of vitamin C is determined by the vitamin C concentration in plasma. In physiological concentrations, ascorbic acid preferentially exerts its antioxidant functions, which help restore cellular 2-oxoglutarate and Fe^2+^ dependent dioxygenase activity (2-OGDDs) [8]. On the other hand, a higher level of ascorbic acid is linked to pro-oxidative functions [7].

## 2. Vitamin C and Its Role in 2-OGDD Enzymes Activity

**Figure 2:**
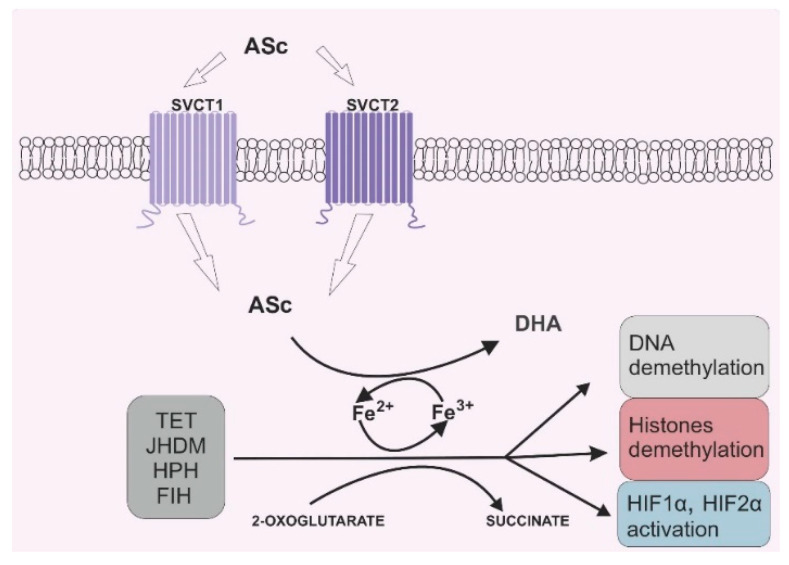
Vitamin C’s role in 2-oxoglutarate and Fe^2+^ dependent dioxygenase (2-OGDD) activity. Legend abbreviations: ASc—ascorbate; SCVT1—sodium dependent vitamin C transporter 1; SCVT2—sodium dependent vitamin C transporter 2; DHA—dehydroascorbic acid, TET—ten–eleven translocation proteins; JHDM—Jumonji C-domain-containing histone demethylases; HPH—hypoxia-inducible factor prolyl hydroxylases; FIH—factor-inhibiting hypoxia-inducible factor; HIF1α—subunit α of the hypoxia inducible factor 1; HIF2 α—subunit α of the hypoxia inducible factor 2. Ascorbate can be engaged in restoring the activity of 2-OGDD enzymes by reducing Fe^3+^ to Fe^2+^, which can be found in the catalytic center of those enzymes. 2-OGDD enzymes also require 2-oxoglutarate to maintain their catalytic activity. TET, JHDM, HPH and FIH are members of the 2-OGDD family, and their main roles in cell biochemistry are based on DNA demethylation (TET proteins), histone demethylation (JHDM proteins) and HIF1α and HIF2 α activation (HPH and FIH proteins). A more detailed description is given in the text.

Due to its antioxidant potential, vitamin C can also serve as an enzyme cofactor. 2-OGDD enzymes require 2-oxoglutarate (2-OG) and Fe^2+^ to maintain their catalytic activity. However, most 2-OGGDs also require ascorbate as a cofactor [9]. The family of 2-OGDDs consists of more than 60 enzymes that contain specific double-stranded β-helix (DSBH) motifs with Fe^2+^ ions in their cores. Their catalytic functions relate to the hydroxylation of particular substrates with the decarboxylation of 2-OG to succinate in the presence of oxygen [10]. Ascorbic acid crucially increases the rate of the reaction catalyzed by 2-OGGD by targeting its catalytic domain and regenerating iron ions from Fe^3+^ to Fe^2+^ (Figure 2) [11].

Research over the past decade has highlighted that vitamin C has a crucial function in epigenetics. In 2009, Tahiliani et al. shed new light on the epigenetic field. The authors discovered that ten-eleven-translocation enzymes (TET: TET1, TET2, TET3), originally considered as chromosomal translocations of the genes MLL and LCX (t(10;11)(q22;q23)) in acute myeloid leukemia patients [12,13], are actually responsible for changing the DNA methylation pattern [14]. Their research indicated that TETs have the ability to induce the hydroxylation of 5-methylcytosine (5-mC) to 5-hydroxymethylcytosine (5-hmC), which leads to DNA demethylation [14]. Later developments in this field revealed that TETs are also involved in the further hydroxylation of 5-hmC to the other cytosine derivatives, which can be subsequently excised by DNA glycosylases [15]. A number of studies have found that ascorbate is an important compound for TET protein activity, since these proteins belong to the 2-OGDD superfamily. It has been proven that ascorbic acid enhances 5-hmC generation by promoting the hydroxylation of 5-mC provided by TET proteins [16]. Additionally, it was conclusively demonstrated that ascorbic acid’s impact on TET proteins is not only due to its reducing ability, as other strong oxidizing agents do not present a similar activity [17]. In line with this finding, vitamin C was proposed to be an agent that promotes DNA demethylation [16,17]. DNA methylation and demethylation are not only linked with cytosine modifications, but also with chromatin reorganization through changes in the amino acids in histones. The enzymes responsible for histone demethylation are Jumonji C-domain-containing histone demethylases (JHDMs), which also belong to the 2-OGDD superfamily [18]. As mentioned in the literature review, more than 20 JHDMs are capable of eliminating the methyl groups of lysines, which are located in histones [9,19]. Previous studies identified that the methylation of lysines can induce or inhibit transcription depending on their location in the histones. Methylation located at lysine 9 in histone H3 and at lysine 20 in histone H4 provides gene silencing, whereas the methylation of lysine 4, lysine 36, and lysine 79 in histone H3 is linked with enhancing transcription [20,21]. A large and growing body of evidence has investigated the essential role of the impaired balance between methylation and demethylation during cancer initiation and progression [22,23,24,25]. In recent years, there has been an increasing interest in TET or JHDM mutations in different cancers, especially hematological ones [26,27,28]. Therefore, the plausible effects of vitamin C in cancer therapy have been the central aim of numerous works of research. 

Other members of the 2-OGDD family are the hypoxia-inducible factors prolyl hydroxylases (HPHs) and the factor-inhibiting hypoxia-inducible factor (FIH), which are arranged during the initiation of the degradation of subunit α of the hypoxia inducible factors HIF1 and HIF2. HIFs take part in cellular adaptation to an anaerobic state, which frequently occurs in tumors [29]. The tumor microenvironment is distinct from that observed in healthy tissues. Tumor cells have the ability to divide very quickly and frequently. Thus, boosting the tumor mass generates separation from blood vessels. Although angiogenesis is common in cancer development, the newly formed vessels are distinct from healthy ones in terms of their structures and functions [30]. Taken together, there is a decrease in the partial pressure of oxygen in tumor masses compared to the pressure observed in corresponding healthy tissue [31,32]. A hypoxic state in tumors can increase the tumor’s invasiveness and ability to metastasize [33]. HIFs are composed of two constitutively expressed subunits, α and β. In normoxic conditions, subunit α is hydroxylated by HPH and FIH; after a multistage post-translational process, subunit α is degraded by proteasomes [29]. However, in a hypoxic state, subunit α is stabilized, followed by bonding with the β subunit, which eventually facilitates the transcription of specific genes. HIF1-α and HIF2-α differ from each other via transcription regulation: HIF1-α is responsible mainly for the regulation of genes involved in cellular metabolic changes, and HIF2-α is responsible for the control of genes involved in cellular signaling and extracellular matrix remodeling factors [34,35,36]. Moreover, both factors exhibit different expressions during hypoxia: in an acute hypoxic state, HIF1-α is highly expressed, whereas long-termed hypoxia results in HIF2-α accumulation [37]. Given that subunit α stabilization is crucial for the expression of HIFs, HPHs and FIH are major factors contributing to cell adaptation under an anaerobic state. Hence, any dysregulation of these enzymes may itself be a trigger of cancer initiation. 

## 3. Vitamin C Transporters: Their Function and Distribution in the Human Body

In the late 1970s, Cameron et al. published a study featuring the significant recovery of cancer patients who ingested vitamin C (10 g orally and 10 g intravenously) [38]. Several years later, two independent randomized controlled studies demonstrated that a daily oral intake of 10 g of ascorbate by patients with cancer resulted in no significant differences from those treated with a placebo [39,40]. However, all these studies suffer from some serious limitations. The main omissions in the above-mentioned research are the routes of administration for vitamin C and the vitamin’s pharmacokinetics in the human body. Preliminary comprehensive work on the relationship between doses of ascorbic acid intake and its concentration in plasma and tissues, as well as bioavailability and urinary excretion, was undertaken by Levine et al. [41]. Based on the results from seven healthy volunteers, the authors determined that the daily recommended dietary allowance for vitamin C is 200 mg/day, but the dose that induces urinary excretion of ascorbic acid is lower (100 mg/day). Moreover, doses higher than 500 mg/day seemed to be ineffective, resulting in nearly complete urinary excretion. Furthermore, 100 mg of vitamin C administered daily was responsible for approximately 60 μM plasma ascorbic acid concentration, and, more importantly, higher oral intake produced plasma concentrations up to 75–90 μM [41]. This finding highlights the unambiguous relationship between the oral administration of ascorbic acid and the upper saturation of the plasma ascorbate concentration. These outcomes further support the tight control of ascorbic acid plasma concentration driven by renal reabsorption and excretion [42,43]. Furthermore, the foregoing studies clearly illustrated that the route of vitamin C administration is a crucial factor for plasma concentration. It was thoroughly demonstrated that the intravenous intake of ascorbic acid can contribute to a 30–60-fold increase in plasma concentration compared to the upper level of its oral doses [41,42,43].

Since vitamin C exhibits a complicated pharmacokinetic range from its absorption to its elimination, its metabolism should be conducted by specialized transporters that regulate it through active or passive transport mechanisms.

Ascorbic acid administration is followed by its absorption in the digestive tract, accumulation in tissues, and reabsorption and excretion by the kidneys. This can be achieved through active transport across cell membranes via the sodium-dependent vitamin C transporters SVCT1 and SVCT2 which were cloned for the first time from rats [44]. SVCTs can actively transport ascorbic acid against the gradient by coupling its entry with sodium inflow to the cell, thus maintaining the sodium gradient throughout the plasma membrane, which is provided by Na/K- ATPase [44,45,46]. However, SVCT2, unlike SVCT1, requires specific ions (Ca^2+^/Mg^2+^) to fully maintain its biological efficiency [46]. Both SVCT1 and SVCT2 display a high affinity to the non-transformed form of ascorbic acid, whereas no other form of vitamin C can be used as a substrate [44,47]. Each transporter is a product of a different gene, either *SLC23A1* or *SLC23A2*. *SLC23A1* is located in chromosome 5 (locus 5q31.2–31.3), whereas *SLC23A2* is mapped to chromosome 20 (locus 20p12.2–12.3). Although both enzymes exhibit 66% amino acid sequence identity, they feature distinctive tissue distributions [48,49]. Interestingly, a review by Bürzle et al. identified a third transporter, SVTC3, which is an orphan transporter whose functions remain unclear. However, this transporter exhibits approximately 30% sequence identity with SVCT1 and SVCT2 [50]. Further work is required to establish SVCT3′s function. Thus far, many studies have been published on SVCT1 and SVCT2. SVCT1 is highly expressed in the epithelium of the small intestine, liver, pancreas, kidneys, reproductive organs, lungs, and skin [44,51]. Both SVCT1 and SVCT2 are found in the intestines. However, the expression of SVCT1 is greater than that of SVCT2 [44]. The most crucial location of SVCT1 is the brush-border membrane of the proximal tubule in renal tissue, which is involved in the renal reabsorption of ascorbic acid. Consequently, SVCT1 is primarily involved in maintaining the vitamin C level in the human body [52,53]. Therefore, a knockout of the *Slc23a1* gene in mice provides almost complete vitamin C urinary elimination, thereby losing approximately 70% of ascorbate tissue supplies [52]. Consequently, the loss of SVCT1 expression in primates may lead to more serious consequences, as they cannot synthetize ascorbic acid by themselves. Moreover, research concerning ascorbic acid transport affinity and capacity underline the key role of SVCT1 in ascorbate reabsorption in the kidneys: SVCT1′s affinity to ascorbic acid is low, but SVCT1 can transport this compound with a high capacity [44,47]. By contrast, the other vitamin C transporter, SVCT2, transports vitamin C with high affinity but with a relatively low capacity [47]. SVCT2 is expressed in almost every cell in the human body, particularly in cells that accumulate vitamin C, such as those in the eyes, adrenal glands, and brain [44,51]. Sotiriou et al. conducted a study in which the authors applied a genetic knockout of the *Slc23a2* gene in a murine model. Prenatal supplementation of ascorbic acid in mice lacking an SVCT2 analog did not have any effect, and the mice died within several minutes after birth due to brain hemorrhage [54]. Thus, it is believed that SCVT2 is essential for brain development. In line with this research, Parker et al. indicated that the pericytes found in brain microvessels are enriched with SVCT2 transporters, which allows such microvessels to collect ascorbate [55].

Since vitamin C easily oxidizes and transforms into DHA upon a pH change, such a change does not inhibit vitamin C transport. DHA transport can be mediated via facilitated diffusion lead by glucose transporters (GLUTs), which is a sodium-independent process. Moreover, the reduced form of vitamin C (AA) is not transferred via GLUT transporters [56,57,58]. Following diffusion, DHA is rapidly reduced into its AA form with the simultaneous oxidation of glutathione and NADPH [59], which is called ascorbate recycling in the literature [60]. As mentioned in the literature review, the GLUT family consists of 14 members that have been identified in most tissues in the human body. Primarily, these members are responsible for glucose transport between the extracellular space and the cells [61]. Nonetheless, previous studies demonstrated that members of the GLUT family can also transport DHA via GLUT1, GLUT3, and GLUT4 [56,57], but none of these transporters is expressed in enterocytes, where vitamin C is absorbed [58]. GLUT2 and GLUT8 are expressed in the intestines, and these two transporters are putatively involved in DHA transfer [58]. However, according to Corpe et al., the DHA transport by GLUTs may be interrupted by dietary factors, which in turn lead to decreased vitamin C bioavailability [58]. Furthermore, a few GLUTs have a considerably weaker affinity for glucose than for DHA. In line with these observations, several cell culture studies suggest that DHA transport is an alternate, or even principal pathway of vitamin C accumulation [62,63]. However, later research on the murine model indicated that SVCT2 knockout in mice led to their demise, despite normally functioning GLUTs [54]. An important question is whether the same pathways of DHA transport are present in human and mice (or other animal) models. As far as we know, GLUT transporters are diverse in human and murine red blood cells [64], and humans and rats show different expressions of GLUT in the intestines [65].

On the other hand, there are several studies concerning the key role of vitamin C transport provided by GLUT in cancer cells [66,67]. A recent paper by Pena et al. suggested that the vast majority of vitamin C transferred from the extracellular space into cancer cells assumes the form of DHA [66]. The authors reported that cancer cells were able to acquire vitamin C, even if they expressed an abnormal form of SVCT2, by using GLUT transporters and converting DHA to AA inside the cells [66]. This phenomenon is called the bystander effect and was previously thoroughly described [63].

## 4. Effects of Vitamin C on Cancer Cells

Due to the pleiotropic functions of vitamin C, the idea of using this compound as a potential anti-cancer drug is not new. However, the past decade has seen increasingly rapid advances in novel approaches to cancer therapy. There is a general agreement that cancer patients exhibit significantly lower level of vitamin C in their plasma compared to healthy subjects [8,68,69]. Moreover, previous studies conclusively demonstrated that ascorbate deficiency can improve the invasiveness of cancer cells [70,71]. One plausible explanation of vitamin C depletion is oxidative stress in cells and reactive oxygen species (ROS) formation over the course of carcinogenesis. It is widely known that cancer cells exhibit different metabolic processes as a result of the dysfunction of the cellular organelles (mainly the mitochondria). This eventually leads to excessive ROS generation, followed by chronic inflammation [72,73]. Vitamin C acts as a radical scavenger due to its antioxidant ability and plays a pivotal role in ROS elimination, so vitamin C levels may eventually decline when free radicals are excessively generated [74]. Therefore, it was suggested that vitamin C may have great value in cancer therapy. In line with this hypothesis, a considerable amount of literature has been published on vitamin C’s ability to destroy cancer cells in vitro and in vivo, even under pharmacological concentrations [7,75,76]. Moreover, it was also indicated that ascorbate has the ability to inhibit cancer growth [77]. There are several possible ways that vitamin C can exert anti-cancer action. One of them is vitamin C’s pro-oxidant ability, which is revealed mainly at high doses and can be accessed only via intravenous intake. Several studies provided by Chen et al. showed that vitamin C may produce hydrogen peroxide (H_2_O_2_) as an intermediate of ascorbate radical generation in cancer cells [7,75,77]. H_2_O_2_, as one of ROS, plays a key role in maintaining the cellular redox state, it may have an impact on disrupting cancer cells’ metabolism [78]. According to Uetaki et al., the main target for vitamin C’s anti-cancer action is the inhibition of glycolysis via a decrease in NAD [78]. Interestingly, vitamin C cytotoxicity is observed only in tumor cells while omitting normal ones [75]. This difference may be the result of an altered mode of ATP generation in cancer cells: instead of oxidative proliferation, such cells preferentially undergo glycolysis, even in an aerobic state. This process is commonly known as the Warburg effect [79]. Hence, vitamin C is targeted in the process and is mainly responsible for energy production in the tumor cells. Furthermore, some cancer-specific mutations are actually associated with the Warburg effect, as previously demonstrated [80,81]. KRAS or BRAF mutations that occur in colorectal cancer may also contribute to glucose uptake and GLUT1 overexpression [82]. As mentioned, GLUT1 transports DHA, which is reduced to ascorbic acid directly after transferring to the cell [59]. A great amount of GLUT1 in KRAS and BRAF mutant cells can be a target for high doses of vitamin C, which can ultimately lead to ROS generation and cancer cell death [81].

However, abnormal cell functions during carcinogenesis are predisposed to reduced oxygen availability [83], and a hypoxic state in cancer cells may restrain the cytotoxic effect of vitamin C [84]. Therefore, there is a second explanation for the selective attack of vitamin C on cancer cells based on vitamin C’s potential to react with iron ions, which can be found in the catalytic centers of multiple enzymes [3]. According to the hypothesis proposed by Ngo et al., there is an abundance of Fe^2+^ ions in specific cancer microenvironments, which can generate H_2_O_2_ and •OH through a reaction with vitamin C [85]. Moreover, the enrichment of Fe^2+^ inside cancer cells may stimulate the diffusion of H_2_O_2_ from extracellular space, which is an intermediate for vitamin C autooxidation [85]. In both cases, an excessive amount of H_2_O_2_ may lead to cytotoxicity in cancer cells.

A large and growing body of studies have demonstrated that the balance between methylation and demethylation is disturbed in many types of cancers [23,86,87,88]. It was found that 5-mC changes involve 5-hmC reductions in tumors [23,24]. Such alterations are proven to have an impact on the transcription of key genes in the human body, including oncogenes and tumor suppressor genes, which may provoke carcinogenesis [89]. Moreover, it was recently detected that the vitamin C level correlates with 5-hmC [90] and, since 5-hmC is potentially involved in the regulation of gene expression [25], ascorbate may also be involved in this process. Ascorbate is a co-factor of TET proteins, which are members of the 2-OGGD family. Several in vitro [16,91,92,93] and in vivo [94] studies have established that ascorbate has the ability to increase 5-hmC. It is also noteworthy that vitamin C removal in vitro correlates with a remarkable decrease in the 5-hmC level with a parallel increase in the 5-mC level [91]. Furthermore, TET2 mutations often occur in hematological malignancies, with approximately 10% in acute myeloid leukemia, 30% in myelodysplastic syndrome, and 50% in chronic myelomonocytic leukemia [95]. Notably, in the case of TET2 mutations, application of vitamin C has the ability to restore TET2 deficiency and promote DNA demethylation [96]. The relationship between TET2 enzymes and ascorbate therapy in leukemic cells was also detected in a study by Zhao et al. [97], who found that vitamin C administration increased TET2 activity. However, in terms of TET2 expression, a similar effect was not detected [97]. Vitamin C in cancer therapy has other beneficial effects on the suppression of tumor growth and metastasis (discussed below) [92,98,99,100], which may be associated with changes in the transcription and expression of genes caused by changes in DNA methylation. As mentioned above, other enzymes regulating the epigenome that depend upon vitamin C are JHDMs connected with chromatin changes [18]. Several studies have identified vitamin C as a crucial agent contributing to the JHDM-dependent chromatin demethylation that occurs during hematopoiesis and somatic cell reprogramming [101,102,103,104]. Given that DNA methylation changes are the most prevalent among the various epigenetic processes, vitamin C likely has great value in the regulation of gene expression.

## 5. Vitamin C in Adjuvant Cancer Therapy

Vitamin C has been frequently prescribed as a complementary or alternative treatment for cancer patients in previous decades. Following the report that intravenous ascorbic acid administration is safe [105], there has been an increasing amount of literature on implementing ascorbic acid therapy in various cancers. However, one question that needs to be asked is whether vitamin C influences conventional chemotherapy or radiotherapy. Vitamin C seems to be effective in preventing the side effects of chemotherapy and radiotherapy [106,107]. However, the ROS generated during radiation may be eliminated by vitamin C, which can diminish the therapeutic effect of radiotherapy. Similarly, chemotherapeutic agents whose pharmacological actions are based on ROS generation may also be inefficient under vitamin C supplementation [108]. Therefore, there is still a need for further research in this field.

### 5.1. Hematological Malignancies

In vitro studies on leukemia cells proved that ascorbic acid plays an important role in modifying the growth of cancer cells [109]. Moreover, even a pharmacological concentration of ascorbic acid is sufficient to particularly kill the primary cancer cells in blood samples from multiple myeloma patients [110]. A recent in vitro study suggested that vitamin C can contribute to increased chemosensitivity in the lymphoma cell line via the induction of SMAD1 expression, which is frequently silenced by methylation in diffuse large B-cell lymphoma [111]. Moreover, the stage of lymphoma is inversely associated with the vitamin C level [111]. Ascorbic acid was also successfully used as an adjunct therapy for hypomethylating agents in AML patients [97]. Additionally, a recent clinical report supports the beneficial role of the intravenous administration of ascorbate in cases of AML with TET2 mutation [112]. Moreover, the parenteral use of vitamin C in AML with a relapse significantly improves blood cell counts and quality of life [113].

### 5.2. Breast Cancer

Similar to hematological malignancies, vitamin C is decreased in more severe cases of breast cancer [114]. Furthermore, a decreased level of vitamin C in breast tumors is linked with higher HIF-1 pathway activity and a more advanced stage of necrosis [114]. Moreover, recent studies indicated that ascorbic acid can induce apoptosis in breast cancer cell lines. According to the authors, vitamin C is associated with the upregulation of TRAIL, which is proven to be an apoptosis inducer [115]. Ascorbic acid also contributes to suppressing the invasion and migration of breast cancer cell lines by inhibiting the epithelial–mesenchymal transition [116]. Interestingly, in vitro studies on a triple-negative breast cancer cell line demonstrated that the concentration of vitamin C obtainable by oral intake is sufficient to inhibit metastatic activity [100]. Furthermore, in vivo studies indicated that the dietary supplementation of ascorbic acid may reduce the mortality of breast cancer patients [117]. Surprisingly, the dietary intake of ascorbic acid in postmenopausal women may actually enhance the risk of breast carcinogenesis [118].

### 5.3. Melanoma

In vitro studies and murine models indicated that vitamin C inhibits invasion and growth in melanoma cells [70,71,92]. According to Chen et al., ascorbic acid promotes the apoptosis of melanoma cells by stimulating the Bax/Bcl-2 pathway, which leads to the activation of caspases followed by protein degradation and cell death [119]. Moreover, a recent study by Yang et al. suggested that ascorbic acid induces cytotoxicity in melanoma cells in a dose-dependent manner [120]. Only high concentrations of vitamin C are sufficient to induce cell death, whereas at lower concentrations, vitamin C can, in fact, promote invasiveness and tumor growth [120]. Vitamin C also plays an important role in the regulation of the HIF1-α protein under normoxic conditions. However, the expression of HIF1-α in cancer cells is extensive, so it may be conducive to the expression of particular proteins associated with melanoma cell motility and invasion [121]. Moreover, in vitro studies indicated that the melanogenesis process induced in melanoma cells may, in fact, be a trigger for an increase in HIF1-α expression [122]. The supplementation of ascorbic acid in melanoma cell lines contributes to the regulation of HIF1-α activity and accumulation [123,124]. Interestingly, a similar effect was not visible for DHA supplementation. Therefore, it seems possible that the ascorbic acid control of HIF1-α stability is reliant on SVCT activity. Moreover, it has been suggested that HIF1-α may interact with the TET2 protein in the melanoma and glioblastoma cell lines [123]. Supplementation of ascorbic acid in melanoma cell lines with TET2 knockdown resulted in an increase in 5-hmC. In addition, HIF1-α knockdown contributed to enhancing TET2 expression in melanoma and glioblastoma cells [123], which shed new light on the plausible regulation of expression between these two proteins in malignant cells.

### 5.4. Glioma and Glioblastoma

Millimolar doses of sodium ascorbate are also believed to have a relevant impact on the inhibition of glioblastoma cell invasion and viability in both in vitro and in vivo models [125]. However, according to the authors, a specific form of cell death provoked by sodium ascorbate called autoschizis needs further examination before using vitamin C as an adjunctive cancer therapy [125]. Nonetheless, a meta-analysis by Zhou et al. indicated that vitamin C intake may help to diminish the chance of glioma incidence [126]. Moreover, several studies indicated that, in glioma patients, intravenous supplementation may successfully serve as an adjuvant tumor therapy and significantly increase survival, as well as reduce and stabilize the tumor [127,128].

### 5.5. Prostate Cancer

Similar to the aforementioned in vitro studies, the effect of vitamin C on prostate cancer cell lines restrains the proliferation and movement of those cell lines [125]. However, the experimental data from in vivo studies are rather controversial, and there is no general agreement about the effects of ascorbic acid on prostate tumors. The parenteral administration of vitamin C to rats with prostate cancer yielded promising results. The ascorbic acid contributed to tumor suppression and the inhibition of metastasis [98]. Favorable results were also achieved in a meta-analysis comprising over 18 prostate cancer studies, indicating the association between vitamin C and a reduction in prostate cancer incidence [129]. Interestingly, according to The Prostate Cancer and Environment Study, ascorbic acid through dietary administration does not decrease the risk of prostate cancer or lower its aggressiveness at diagnosis [130]. Similar results were obtained in a posttrial study by Wang et al., who indicated that vitamin C supplementation did not contribute to prostate cancer occurrence [131].

## 6. Impaired SVCT Function in Cancer

### 6.1. Polymorphisms of SVCT

The vitamin C concentration in plasma and tissues has been identified as a one of the major contributing factors for cancer incidence. As mentioned above, cancer patients often exhibit a lower level of vitamin C in their plasma [8,68,69]. Apart from ROS generation and a changed redox status in cancer cells, it was suggested that this phenomenon may be linked to the single nucleotide polymorphisms (SNPs) of the vitamin C transporters SVCT1 and SVCT2. SNPs in the coding regions of *SLC23A1* and *SLC23A2* are implicated in the vitamin C level in plasma and its transport inhibition [132,133]. A cohort study involving 15000 participants by Timpson et al. conclusively demonstrated that *SLC23A1* rs33972313 is involved in decreasing of vitamin C circulating concentrations, which decreased by over 4 µmol [132]. According to a murine study by Corpe et al., *SLC23A1* rs33972313 is associated with a 40–50% reduction in ascorbate accumulation in cells [52]. Furthermore, a European Prospective Investigation into Cancer and Nutrition (EPIC) cohort study revealed that both SVCT1 and SVCT2 SNPs are implicated in the vitamin C plasma concentration [133]. This study determined that two *SLC23A1* SNPs, rs11950646 and rs33972313, are involved in vitamin C decreases of 10–13% and 24%, respectively. Interestingly, two *SLC23A2* SNPs, rs6053005 and rs6133175, were both found to be associated with an ascorbic acid increase of 24% [133]. This discrepancy may be due to the different roles and types of localization among vitamin C transporters. Although this subject needs further research, SVCT1 and SVCT2 SNPs may serve as ascorbic acid level predictors.

While this process is still being explored, previous studies reported the involvement of SVCT SNPs in several cancers. Since SVCT1 is responsible for vitamin C absorption and reabsorption, it is possible to find the *SLC23A1* SNP in gastric cancers. Surprisingly, none of the SVCT1 genetic polymorphisms are associated with gastric cancer risk [133,134]. However, polymorphisms of the second vitamin C transporter SVCT2: *SLC23A2* (*SLC23A2* rs6116568 and *SLC23A2* rs12479919) were correlated with gastric cancer incidence [133,134]. The concentration of vitamin C in the gastric juice and mucosa is significantly higher than that observed in plasma [135], which highlights vitamin C’s important role in stomach function. However, previous studies indicated that rat gastric glands are rich in SVCT2 instead of SVCT1 [44]. Thus, SVCT2 alone may play a crucial role in vitamin C absorption.

The involvement of a genetic polymorphism of SVCT1 in cancer was detected in follicular lymphoma. A large population-based case–control study revealed that *SLC23A1* rs6596472 and *SLC23A1* rs11950646 may increase the risk of follicular lymphoma by up to 80% [136]. An elevated risk of follicular lymphoma was also associated with a genetic variant of SVCT2 (*SLC23A2* rs1776948). The same variant and two others (*SLC23A2* rs6133175 and *SLC23A2* rs1715364) were also identified in chronic lymphocytic leukemia (CLL) [136]. A study involving over 400 CLL cases detected a significant correlation between the same two SVCT2 polymorphisms (*SLC23A2* rs6133175 and *SLC23A2* rs1776948) with CLL development [137]. Moreover, these genetic variants of *SLC23A2* could not be modulated by fruit and vegetable intake as a dietary principal source of vitamin C. However, the CLL patients had greater fruit administration than healthy subjects [137]. Dietary vitamin C consumption was also not found to be associated with a risk of advanced colorectal adenoma [138]. However, incidence of this cancer was correlated with two genetic variations of SVCT2, *SLC23A2* rs4987219 and *SLC23A2* rs1110277 [138]. The genetic variant *SLC23A2* rs4987219 was also found to be a possible modifier of human papillomavirus 16 (HPV16)-associated head and neck cancer [139]. According to this study, predispositions for HPV16 infection followed by this type of head and neck cancer are strongly associated with vitamin C metabolism. It was suggested that genetic variations of SVCT transporters have distinct exposures to HPV16 [139]. It cannot be excluded that SNPs found in the *SLC23A1* and *SLC23A2* genes may have an impact on transcription regulation. All known effects of SVCT polymorphisms are collected in Table 1.

### 6.2. Altered Expression of SVCT

The location of SVCT1 is mostly limited to epithelial tissues; hence, SVCT1 is primarily responsible for vitamin C homeostasis and circulation in the whole body [44,51]. Given that a loss of *SLC23A1* contributes to serious vitamin C deficiency in mice [52], it seems possible that alterations in SVCT1 may be far more severe in humans. To date, there is no study on SVCT1 loss in humans. On the other hand, SVCT2 is located in the vast majority of metabolically active tissues and conceivably controls vitamin C accumulation [44,51]. Hence, it seems possible that alterations in SVCT2 may be involved in carcinogenesis. Therefore, several studies analyzed SVCT2 expression. SVCT2 expression has been detected in multiple tumor samples, with a significantly higher percentage of intracellular, rather than membrane, immunoreactivity [67]. Moreover, studies on breast cancer have indicated the differential expression of SVCT2 in breast tumors, with the highest level in hormone-independent breast cancer. Normal breast epithelium samples, however, have not shown SVCT2 expression. Interestingly, the expression of SVCT1 in breast tumor samples has not been detected [66]. Further examination of the breast cancer cell lines revealed that these cell lines are incapable of vitamin C intake in an ascorbic acid form via SVCT transporters and can only intake in ascorbic acid form as DHA using GLUT transporters. This observation indicates SVCT2 deficiency in the cell membrane. However, the expression of this transporter has been identified in the mitochondria, where it is presumably responsible for low affinity vitamin C transport [66]. According to a study by Hong et al., SVCT2 is crucial for ascorbate-induced cancer cell death [140]. Ascorbate transport via SVCT2 to cancer cells is associated with enhanced intracellular ROS generation, which eventually leads to breast cancer cell termination. Furthermore, SVCT2 knockdown in breast cancer cells with previously high SVCT2 expression resulted in resistance to ascorbic acid treatment [140]. The association between SVCT2 expression and ascorbic acid treatment has also been evaluated in colorectal cancer cell lines [141]. Similar to a previous study, the cytotoxicity of ascorbic acid was proportional to the expression of SVCT2. Moreover, the cellular response to ascorbic acid treatment was dependent on SVCT2 expression. Cancer cells with low SVCT2 expression levels exhibited anti-cancer effects at high doses of ascorbic acid and a proliferative effect at low doses of this compound. In contrast, cancer cells with high SVCT2 expression exerted anti-cancer outcomes at all ascorbic acid concentrations. A possible explanation for this discrepancy is the insufficient ROS generation at low ascorbic acid concentrations in colorectal cancer cells with low SVCT2 expression [141]. The intensified cytotoxicity induced by ascorbic acid, which depends on SVCT2 expression, has also been investigated in hepatocellular cancer [142] and cholangiocarcinoma [143]. Moreover, colorectal cancers with KRAS mutations are often resistant to cetuximab, which is a major drug used in the therapy of these cancers [144]. An in vitro study on colon cancer cells with KRAS mutations showed that vitamin C can partner with cetuximab to induce cell death depending on SVCT2 expression [145]. According to the authors, ascorbic acid combined with cetuximab may impact the MAPK/ERK signaling pathway, which is disturbed in colon cancers with a KRAS mutation. However, specific changes in MAPK/ERK signaling after ascorbic acid and cetuximab exposure were observed only in colorectal cancer cells with SVCT2 expression [145]. Furthermore, the impact of ascorbic acid on MAPK/ERK signaling, which depends on SVCT2 expression, was also discovered in mouse neuroblastoma cells [146]. It was suggested that the overexpression of SVCT2 is associated with the differentiated phenotype of N2a mouse neuroblastoma cells. The supplementation of ascorbic acid to neuroblastoma cells with SVCT2 overexpression resulted in the promotion of MAPK/ERK phosphorylation, which may eventually lead to central nervous system development [146].

## 7. Conclusions

Since its conception, the possibility of treating cancer with vitamin C has been a controversial and much-disputed subject. Some published data have indicated promising findings for vitamin C’s role in cancer therapy, as well as its bioavailability in cancers. Its clinical anticancer potential is reflected in the number of clinical trials involving vitamin C. The level of vitamin C in cells may be related to vitamin C transporter expression and its polymorphism in cells. Therefore, mutations of the two main vitamin C transporters may contribute to their impaired function and thus may plausibly contribute to cancer development, as suggested in this review. Further investigations are needed to estimate the actual association between polymorphisms of SVCT, cancer risk and incidence. Additionally, the positions of genetic polymorphisms in the *SLC23A1* and *SLC23A2* genes include intrones and exones [147]. Hence, the reason why polymorphisms located in intrones play a role in vitamin C regulation remains ambiguous. It seems possible that genetic variations may have an impact on the regulation of SVCT transcription. According to Skibola et al., in silico models indicate that the polymorphisms of *SLC23A2* (*SLC23A2* rs1715364) may increase *SLC23A2* expression, which may consequently intensify vitamin C bioavailability [136]. Vitamin C appears to be a promising candidate for cancer prevention and treatment. However, there is still a need for further research, especially on the role of vitamin C transporter polymorphisms in different types of cancers and cancer treatments.

## Figures and Tables

**Table 1:** SVCT polymorphisms and their implications.

SVCT Type	SNP ID	Effect	References
SVCT1	*SLC23A1* rs33972313	decrease in circulating vitamin C	Timpson et al., 2010 [132]
		40–50% reduction in ascorbate accumulation in murine cells	Corpe et al., 2010 [52]
		24% decrease in vitamin C’s plasma concentration	Duell et al., 2013 [133]
	*SLC23A1* rs11950646	10–13% decrease in vitamin C’s plasma concentration	Duell et al., 2013 [133]
	*SLC23A1* rs6596472	higher risk of follicular lymphoma	Skibola et al., 2008 [136]
	*SLC23A1* rs11950646	higher risk of follicular lymphoma	Skibola et al., 2008 [136]
SVCT2	*SLC23A2* rs6053005	24% increase in vitamin C’s plasma concentration	Duell et al., 2013 [133]
	*SLC23A2* rs6133175	24% increase in vitamin C’s plasma concentration	Duell et al., 2013 [133]
	*SLC23A2* rs6116568	higher risk of gastric cancer	Duell et al., 2013 [133]
	*SLC23A2* rs12479919	higher risk of gastric cancer	Wright et al., 2009 [134]
	*SLC23A2* rs1776948	higher risk of follicular lymphoma	Skibola et al., 2008 [136]
		higher risk of chronic lymphocytic leukemia (CLL)	Skibola et al., 2008 [136] Casabonne et al., 2017 [137]
	*SLC23A2* rs6133175	higher risk of chronic lymphocytic leukemia (CLL)	Skibola et al., 2008 [136] Casabonne et al., 2017 [137]
	*SLC23A2* rs1715364	higher risk of chronic lymphocytic leukemia (CLL)	Skibola et al., 2008 [136]
	*SLC23A2* rs4987219	higher risk of colorectal adenoma	Erichsen et al., 2008 [138]
		plausible modifying factor of HPV16- associated head and neck cancer	Chen et al., 2009 [139]
	*SLC23A2* rs1110277	higher risk of colorectal adenoma	Erichsen et al., 2008 [138]
